# MUC1 confers radioresistance in head and neck squamous cell carcinoma (HNSCC) cells

**DOI:** 10.1080/21655979.2020.1791590

**Published:** 2020-07-14

**Authors:** Tian-qiao Huang, Ya-nan Bi, Zheng Cui, Jin-ping Guan, Yi-chuan Huang

**Affiliations:** a Department of Otolaryngology, The Affiliated Hospital of Qingdao University, Qingdao, Shandong, China; b Operating Room, The Affiliated Hospital of Qingdao University, Qingdao, Shandong, China; c Endoscopy, The Affiliated Hospital of Qingdao University, Qingdao, Shandong, China; d Emergency Surgery, The Affiliated Hospital of Qingdao University, Qingdao, Shandong, China

**Keywords:** Head and neck squamous cell carcinoma, radioresistance, X-ray irradiation, DNA double-strand break, MUC1

## Abstract

Mucin 1 (MUC1), a transmembrane glycoprotein, has shown to be as the possible prognostic marker to predict the risk of aggressive head and neck squamous cell carcinoma (HNSCC). In the present study, we investigated the effect of MUC1 in HNSCC cells and the response to X-ray irradiation (IR). Here, we examined the impact of MUC1 overexpression or downexpression on clonogenic survival and apoptosis in response to X-ray irradiation (IR). Radioresistance and radiosensitivity were also observed in HNSCC cells that are MUC1 overexpression and MUC1 downexpression. This enhanced resistance to IR in MUC1-overexpressing cells is primarily due to increased the number of radiation-induced γH2AX/53BP1-positive foci and DNA double-strand break (DSB) repair kinetics. MUC1 overexpression repaired more than 90% of DSBs after 2 Gy radiation by 24 h compared to the empty vector overexpressing cells with less than 50% of DSB repair. However, MUC1 downexpression repaired less than 20% of DSBs compared to the empty vector-overexpresing cells. MUC1 overexpression inhibited proapoptotic protein expression, such as caspase-3, caspase-8, and caspase-9, and induced antiapoptotic protein Bcl-2, followed by resistance to IR-induced apoptosis. Our results showed that targeting MUC1 may be as a promising strategy to counteract radiation resistance of HNSCC cells.

## Introduction

Head and neck squamous cell carcinoma (HNSCC) originates from the mucosa of the oral cavity, pharynx, and larynx, which is characterized by poor survival rates in advanced stages and fails current treatments. Treatment consists of surgery and/or radiation therapy, with the addition of chemotherapy for patients with advanced disease [1]. However, the combined therapy in HNSCC patients with unresectable advanced disease only achieved less than <10% 5-year survival rate of [2]. Part of the reason is that resistance to chemo- and radiotherapy often occurs. In order to overcome these radioresistance, the development of an effective strategy is desired for the treatment of HNSCC.

It has recently found that targeting radioresistant tumor cells is essential for enhancing the efficacy of radiotherapy; however, the activated signals in resistant tumors are still unknown. Though several factors, such as overexpression of EphA3, PAG1, or type 1 IGF receptor, and GLUT-1 [3,4] are reportedly activated and related to the radioresistance of HNSCC, targeting these activated genes does not completely reverse radioresistance. Therefore, the detailed mechanism by which the radioresistance of HNSCC is induced still unknown.

Mucin 1 (MUC1) is a transmembrane glycoprotein which is expressed in normal epithelial cells, but it was overexpressed and aberrantly glycosylated in majority of carcinomas [5], including laryngeal cancer [6,7]. Accumulating evidence supported the involvement of the oncogenic MUC1 in tumor metastasis. MUC1 overexpression did not relate to survival but correlate with T stage, and advanced T stage was associated with prognosis [7]. Numerous studies demonstrated that MUC1 confers resistance to apoptosis or necrosis in the response of cells to DNA damage [8,9], reactive oxygen species (ROS)-induced stress [10,11] and hypoxia [12,13]. MUC1-mediated protection against irradiation-induced apoptosis was also through activation of the JAK2/STAT3 signaling pathway, and induction of antiapoptotic proteins Mcl-1 and Bcl-xL [14]. Elevation of miR-512-5p contributes to the reduction of radioresistance in cervical cancer cells by inhibiting MUC1 expression [15]. Gunda et al. [16] reported that MUC1-mediated nucleotide metabolism plays a key role in facilitating radiation-resistance in pancreatic cancer and targeted effectively through glycolytic inhibition. These data indicated that MUC1 overexpression contributes to chemoresistance of cervical cancer cells. MUC1 is definitely expressed in laryngeal squamous cell carcinoma (SCC), and no significant difference of MUC1 expression was found between SCC as compared to normal larynx [17]. Kang et al. [18] have already reported that X–irradiation induced MUC1 expression in cultured human colon carcinoma HT-29 cells and resistant to X–irradiation treatment. However, whether MUC1 overexpression contributes to radioresistance in HNSCC cells is still unclear.

In the study, we performed loss-or gain-of-function approaches in HNSCC cell lines to investigate the contribution of MUC1 in regulation of IR-induced cell survival and apoptosis. We demonstrate that targeting MUC1 inhibited cell growth and induced apoptosis and renders HNSCC cells sensitive to IR treatment *in vitro*. In the study, we aimed to analyze the impact of MUC1 on radiation-induced DNA damage repair and explored its mechanisms.

## Materials and methods

### Cell culture

Human HNSCC cell line Hep2 was obtained from CoBioer (Nanjing), and the TU-212 cell line was obtained from XINYU BIOLOGICAL TECHNOLOGY CO., LTD. (Shanghai). TU686, TU177, AMC-HN-8, and 293T cells were purchased from BNBIO (Beijing, China), and cultured in RPMI-1640 medium supplemented with 10% fetal bovine serum or Dulbecco’s modified Eagle’s medium supplemented with 10% FBS.

### Antibodies

Monoclonal antibodies anti-phospho-Histone γH2AX (Ser139) and 53BP1 were purchased from Cell Signaling Technology, Inc. (Shanghai, China); Anti-cleaved-caspase-3, -8, -9, and caspase-3, -8, -9, bcl-2 monoclonal antibodies, and the horseradish peroxidase-conjugated secondary antibodies were purchased from Santa Cruz Biotechnology, Inc (Shanghai, China). Monoclonal anti-b-actin was purchased from Sigma-Aldrich (St. Louis, MO). Fluorescent dye-conjugated goat anti-rabbit IgG was obtained from Invitrogen Corp (Shanghai, China).

### Construction of the recombinant lentivirus and lentivirus infection

The short hairpin oligonucleotides MUC1 shRNA and control shRNA (NC shRNA) were synthesized and cloned into the lentivirus‐based vector pGP‐L (SBI, Palo Alto, CA, USA). Lentivirus production was followed as a standard protocol by transfecting 293T cells with recombinational shRNA vector, together with pVSVG‐I and pCMVΔR8.92 (Sigma, St. Louis, MO, USA). For cell transfection, TU686 cells were cultured in 6‐well plates and transfected with the MUC1 shRNA and NC shRNA. Puromycin 1.5 μg/ml (Gibco life technologies, Germany) was used to select stable clones for 1 week.

### MUC1 plasmid transfection

Hep2 cells were transfected with either the pcDNA3.1 (NC) or pcDNA3.1-MUC1 (MUC1) plasmids using FuGene HD Transfection Reagent (Promega, Germany) according to the manufacturer’s instruction. Following selection with 0.4 mg/ml Neomycin (Calbiochem Merck, Germany) for 2 weeks, stable single clones were isolated and MUC1 expression was confirmed on by Western blot and RT-PCR assay.

### Cell irradiation

Cells were irradiated with 0, 2, 4, 6, and 8 Gy IR, respectively, plated in serum-free medium containing 1% penicillin–streptomycin and incubated at 37°C for 24–72 h. Then, the cells were washed with PBS for further study.

### Colony formation assay

The stable vehicle (NC) or MUC1 transfected Hep2 cells or stable vehicle shRNA (NC shRNA) or MUC1 shRNA transfected TU686 cells were trypsinized, counted, and the appropriate number of cells was plated in 60-mm dishes and allowed to attach for 24 h. After 24 h, the cells were irradiated (2–8 Gy) and incubated for 8–10 days. The colonies were stained with crystal violet (Sigma Chemical Co.), and colonies of 50 cells or greater were counted.

### MTT assay

The stable vehicle (NC) or MUC1 transfected Hep2 cells or stable vehicle shRNA (NC shRNA) or MUC1 shRNA transfected TU686 cells were seeded into a medium containing 10 ml of fresh medium at a concentration of 1 × 10^3^ cells/ml in a 96-well microplate, after 24 h, cells were irradiated with 0, 2, 4, 6, or 8 Gy γ-irradiation. Cells were allowed to grow for an additional 72 h and cell viability was analyzed using the 3-(4,5-Methylthiazol-2-yl)-2,5-diphenyl-tetrazolium bromide (MTT, Applichem) assay, according to the manufacturer’s protocol. The reaction product was measured on a microtiter plate reader (Wallac VICTOR 1420, PerkinElmer, Waltham, USA) with a reference wavelength of 490 nm.

### Apoptosis assay

Apoptosis was analyzed by flow cytometer-based Annexin V/PI method. Briefly, cells were seeded in 96-well plates at a density of 3000 cells per well. All the cells in each group were harvested at 72 h after 2–8 Gy irradiation. Cells were trypsinized and harvested by centrifugation and then incubated with Annexin V and PI for 15 min at room temperature. Apoptosis was examined by flow cytometry using Annexin V-FITC/PI kit (Becton Dickinson, Mountain View, CA, USA).

### Western blotting

Cells were lysed in phosphate-buffered saline (PBS) at 4°C for 30 s. Lysates were clarified by centrifugation 10 000 rpm (9391 × *g*), and proteins were separated on 4% to 12% SDS-polyacrylamide gels. Briefly, 30–50 μg of protein was resolved by SDS-PAGE, transferred to PVDF membranes. The membrane was blocked with 5% skim milk in Tris-buffered saline with Tween (TBS-T) solution for 30 minutes at room temperature and incubated with a diluted solution of primary antibody. The primary antibodies were anti-MUC1 (1:500 dilution, Cell Signaling Technology, Shanghai, China), anti-bcl-2 (1:1000 dilution, Abcam, Shanghai, China), β-actin (1:1000 dilution, Abcam, Shanghai, China); anti‐cleaved‐caspase-3, -8, -9 (1:500 dilution, Abcam, Shanghai, China.) All primary antibodies were incubated overnight at 4°C and detected using anti-mouse- or anti-rabbit immunoglobulin horseradish peroxidase (HRP)-conjugated antibodies (Southern Biotech), and the blots bands were visualized with an enhanced chemiluminescence system (AmershamBioscience). Densitometric analysis of Western blots was performed using VisionWorks LS, version 6.7.1.

### Immunofluorescence microscopy

Transfected or non-transfected cells were cultured on poly–lysine-coated cover slips and exposed to total dose of 2 Gy radiation. Then, the cells were fixed with 3% paraformaldehyde and 2% sucrose (pH 7.3) and washed with ice-cold PBS and blocks with PBS containing 0.1% Triton X-100, 0.1% BSA (Sigma), and 3% donkey serum (Sigma). The labeling was performed with mouse anti–phospho-Histone γH2AX, anti-53BP1, Alexa Fluor 488-conjugated goat anti-Rabbit, and rhodamine red–conjugated goat anti-mouse. DAPI was used to stain nuclear DNA. Cells were viewed and imaged using 60× oil immersion lens on an Olympus BX51 fluorescence microscope and were further processed by ImageJ software.

### Statistical analysis

The results are presented as means of three independent experiments ± SEM. Analyses were performed using SPSS.22 soft by Student’s *t*-test or Chi-square where indicated. p values < 0.05 were considered to indicate statistical significance.

## Results

### Silencing of MUC1 with shRNA in TU686 cell lines

**Figure 1.**
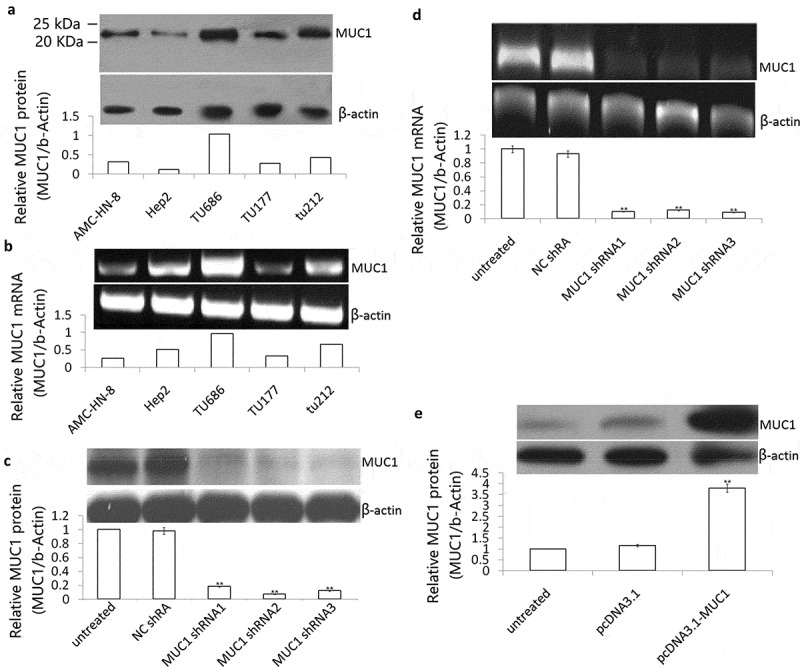
Effect of shRNA or plasmid transfection on MUC1 expression in LSCC cells. (a) MUC1 protein expression was detected in Hep2, TU-212, TU686, TU177 and AMC-HN-8 cells by Western blot assay; (b) MUC1 mRNA was detected in Hep2, TU-212, TU686, TU177 and AMC-HN-8 cells by RT-PCR assay; (c) TU686 cells were transfected into MUC1 shRNA 1, 2, and 3 for 72 h, MUC1 protein expression was detected by Western blot assay; (d) MUC1 mRNA expression was detected by RT-PCR assay; E, Hep2 cells were transfected into pcDNA3.1 or pcDNA3.1 MUC1 for 72 h, MUC1 protein expression was detected by Western blot assay. vs untreated.**P < 0.01.

MUC1 expression was monitored on transcript and protein levels in well-established HNSCC cell line Hep2, TU-212, TU686, TU177, and AMC-HN-8 cells. MUC1 protein was detected in all 5 HNSCC cell lines, with the highest levels in **TU686** cells, and lowest levels in Hep2 cells by Western blot assay (Figure 1(a)). MUC1 transcripts were also evident on mRNA level by RT-PCR analysis (Figure 1(b)).

MUC1 shRNA was transfected successfully into the **TU686** cells, and the knockdown effect was verified by qPCR and immunoblotting. The expression of MUC1 at the mRNA and protein levels significantly decreased after treatment with the shRNA for 72 h (*P* < 0.01, Figure 1(c, d)). These data indicated a successful silencing of *MUC1* gene expression induced by the shRNA in **TU686** cells.

### Enforced MUC1 expression in Hep2 cell lines

MUC1 was transfected successfully into the Hep2 cells, and the overexpressed effect was verified by immunoblotting. The expression of MUC1 at protein levels significantly increased after treatment with the MUC1 for 72 h (*P* < 0.01, Figure 1(e)). These data indicated a successful transfection of *MUC1* gene expression induced by the pcDNA3.1-MUC1-Myc/His in the Hep2 cells.

### Increased MUC1 expression enhances radioresistance

**Figure 2.**
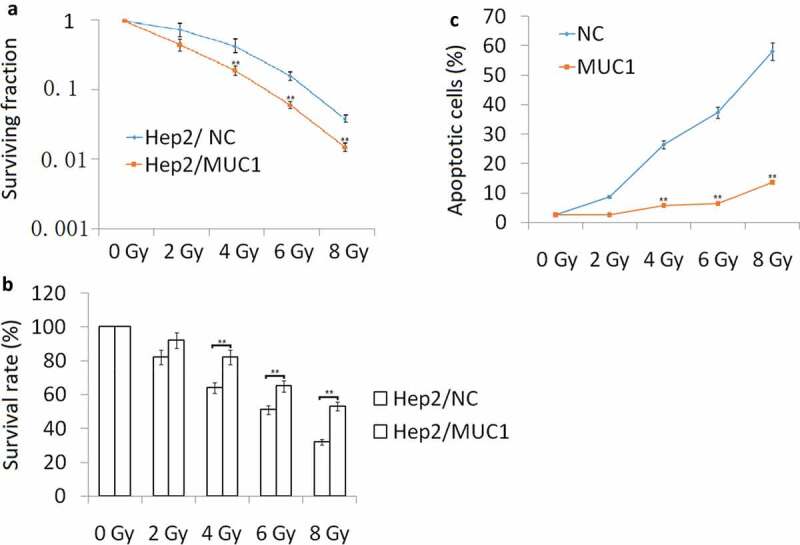
Increased MUC1 expression increases radioresistance in Hep2 cells. (a) Clonogenic survival of Hep2/MUC1 and Hep2/NC. (b) Viability assay of Hep2/MUC1 and Hep2/NC cells in response to radiation; (c) Cell apoptosis was detected by flow cytometry assay. **P < 0.01.

Hep2 cells were transfected with MUC1 for 72 h, then exposure to 2, 4, 6, 8 Gy IR for 2 h. Our data showed that MUC1 transfected Hep2 cells showed higher radioresistance compared with vehicle (NC) Hep2 cells by Clone formation analysis (Figure 2(a)), MTT assay (Figure 2(b)), and cell apoptosis assay following increasing doses of IR (Figure 2(c)). To further characterize apoptosis in Hep2 cells, Western blot analysis was done (Figure 4(a)). The cleaved-caspase-3, cleaved-caspase-9, and cleaved-caspase-8 were significantly higher in NC transfected followed by IR treatment for 24 h than MUC1 transfected Hep2 cells, whereas the antiapoptotic protein Bcl-2 levels were decreased in NC transfected followed by 2–8 Gy IR for 24 h than MUC1 transfected Hep2 cells. These data clearly demonstrate that MUC1 overexpression significantly contributes to the development of radioresistance in HNSCC cells by reducing expression of proapoptotic proteins.

### *Decreased MUC1 expression enhances radiosensitivity* in vitro

**Figure 3.**
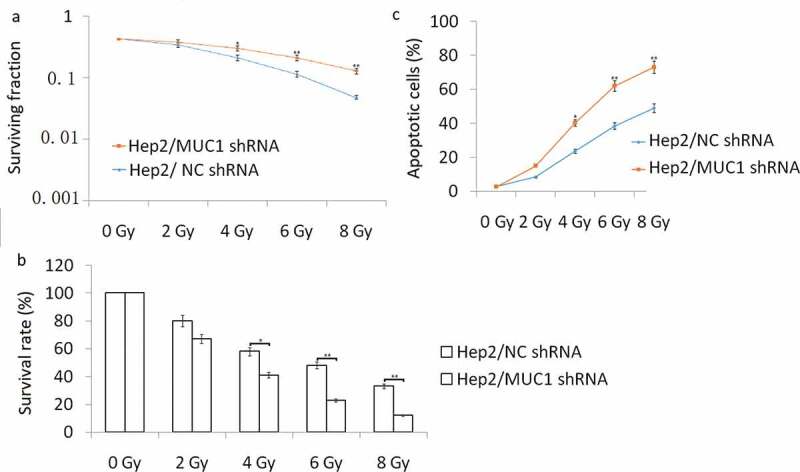
Decreased MUC1 expression increases radiosensitivity in TU686 cells. (a) Conogenic survival of MUC1 shRNA and NC shRNA transfected cells. (b) Viability assay of MUC1 shRNA and NC shRNA cells in response to radiation. (c) Cell apoptosis was detected by flow cytometry assay. *P < 0.05;**P < 0.01.

**Figure 4.**
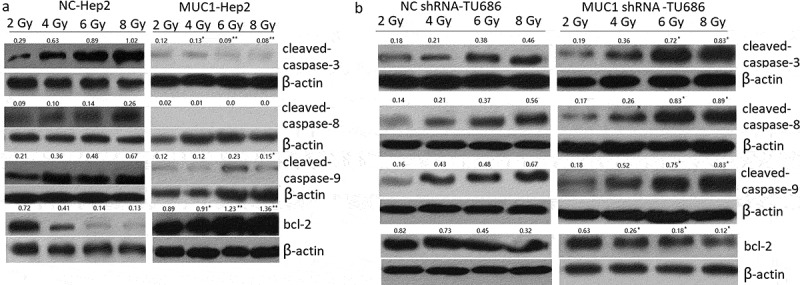
Proapoptotic and antiapoptotic proteins expression in shRNA or plasmid-transfected LSCC cells. (a) Western blot analysis of proapoptotic and antiapoptotic proteins in MUC1 and NC cells in response to radiation. (b) Western blot analysis of proapoptotic and antiapoptotic proteins in MUC1 shRNA and NC shRNA cells in response to radiation. *P < 0.05;**P < 0.01.

We next investigated whether targeting MUC1 enhances the sensitivity of **TU686** cells to IR treatment. **TU686** cells were transfected with MUC1 shRNA for 72 h, following by 0–8 Gy IR treatment and then subjected to clonogenic, cell viability, and apoptosis assays. The results showed that targeting MUC1 enhanced IR-induced cell growth inhibition and cell apoptosis compared to empty vector transfected **TU686** cells by colony formation assay (Figure 3(a)), MTT assay (Figure 3(b)) and flow cytometry analyses (Figure 3(c)).To further characterize apoptosis in **TU686** cells, Western blot analysis showed that the cleaved- caspase-3, cleaved- caspase-9, and cleaved- caspase-8 were increased in MUC1 shRNA transfected followed by 2–8 Gy IR for 24 h than NC shRNA transfected **TU686** cells, whereas the antiapoptotic protein Bcl-2 expression was decreased in MUC1 shRNA transfected **TU686** cells followed by 2–8 Gy IR for 24 h than NC shRNA transfected **TU686** cells (Figure 4(b)). These data indicated that MUC1 overexpression increased radioresistance in HNSCC cells by enhancing expression of proapoptotic proteins.

### Enhanced MUC1 expression induced IR resistance via enhancing DSB repair

**Figure 5.**
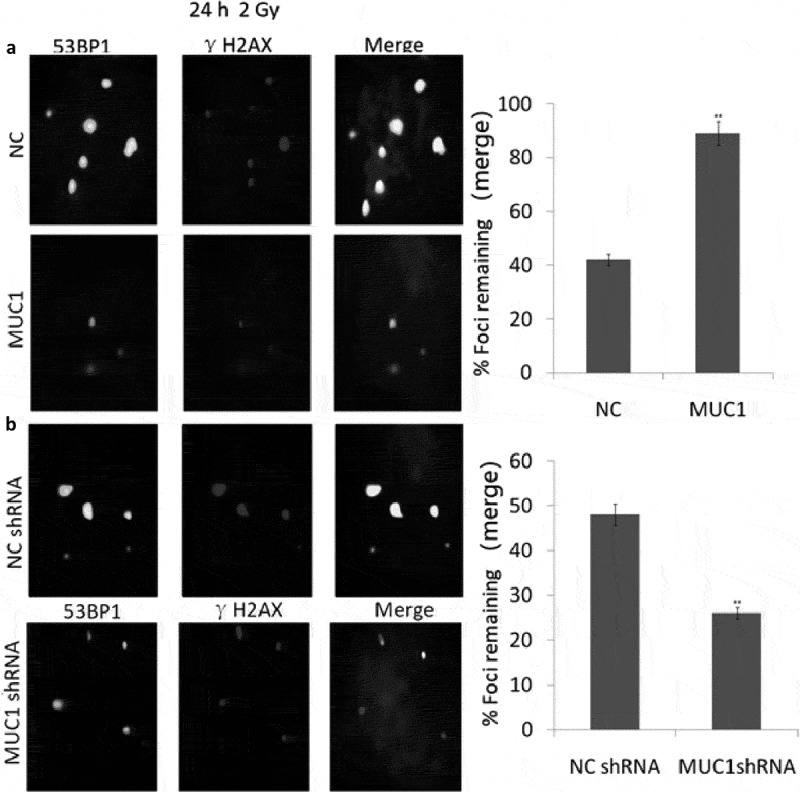
Accelerated DSB repair in MUC1-overexpressing cells after IR. (a) NC and MUC1 transfected cells were irradiated with 2 Gy and immunostained for 53BP1 (green) and phospho-γH2AX (red) foci at 24 h after radiation. (b) NC shRNA and MUC1 shRNA transfected cells were irradiated with 2 Gy and immunostained for 53BP1 (green) and phospho-γH2AX (red) foci at 24 h after radiation. Colocalized foci (yellow) were counted at 24 h (average, 50 nuclei). DNA repair kinetics between these two cells was obtained by plotting the percentage of remaining foci against time.

The MUC1-overexpressing and Vector-expressing cells were exposed to a total dose of 2 Gy and samples were collected at 24 h. 53BP1 and phospho-γH2AX foci were detected via dual immunofluorescence staining, and DSB repair kinetics was determined by counting the colocalized foci in the cells. The results showed that MUC1-overexpression results in significantly faster rate of DSB repair compared with the empty vector transfected cells (Figure 5(a)). Furthermore, 90% repair was completed at 24 h in the MUC1-overexpressing cells, and 90% repair empty vector transfected cells following IR at 24 h. These data indicate that enhanced MUC1 expression accelerated DSB repair kinetics and modulates radioresistance.

### Targeting MUC1 enhanced IR sensitivity via inhibiting DSB repair

MUC1 shRNA and NC shRNA transfected TU686 cells were exposed to a total dose of 2 Gy and samples were collected at 24 h. The results showed that targeting MUC1 by MUC1 shRNA inhibited DSB repair (Figure 5(b)). Less than 20% repair was completed at 24 h in the MUC1 shRNA transfected TU686 cells, compared with the NC shRNA transfected cells with 45% repair following IR at 24. These data indicate that targeting MUC1 is associated with decreased DSB repair kinetics and radiosensitivity.

## Discussion

Radiotherapy is a mainstay for the treatment of many human cancer types, including head and neck squamous cell carcinoma (HNSCC). However, radioresistance-induced recurrence is the primary cause of HNSCC treatment failure. Unfortunately, there is no available treatment specific to HNSCC patients with radioresistance. MUC1 is a potential tumor promoter gene and plays an important role in regulating cell invasion, proliferation, survival, and apoptosis [19,20]. MUC1 overexpression is often observed in HNSCC cells [6,7] and is associated with poor prognosis [6]. A similar relationship between MUC1 expression and aggressive tumor is also reported in duodenal adenocarcinoma [21] and esophageal adenocarcinoma [22]. MUC1 overexpression in multiple solid tumors reduces overall survival and imparts resistance to radiation and chemotherapies [16,23–26]. Nevertheless, this is the first study to report that the MUC1 overexpression in HNSCC cells contributes to the resistance to IR.

Apoptosis is an important mechanism by which IR exerts its therapeutic response and faulty apoptosis is a known mechanism leading to resistance to radiation therapy [27]. In the present study, IR treatment induced significant cell apoptosis, however, enforced MUC1 expression inhibited IR-induced cell apoptosis, and targeting MUC1 accelerated IR-induced cell apoptosis *in vitro*. The results support the observation that MUC1-overexpressing cells are resistant to IR-induced apoptosis, and MUC1-downexpressing cells are sensitive to IR-induced apoptosis. In an additional experiment MTT and colony formation assay, we monitored the MUC1 on IR-induced cell growth *in vitro*, only to found that silencing MUC1 reduced IR-induced cell growth, and vice versa. These results suggest that the MUC1 overexpression is partially responsible for the development of radioresistance and that the MUC1 silencing is a potential strategy to overcome radioresistance in HNSCC.

It is known that caspase family and Bcl-2 family are the major families involved in apoptosis [28]. Caspase-8 is associated with activating ‘extrinsic’ and ‘intrinsic’ apoptosis pathway, and caspase-3 and caspase-9 are associated with both ‘extrinsic’ and ‘intrinsic’ apoptosis process. However, bcl-2 is the key antiapoptotic protein. In the study, we compared the expression of caspase-3, caspase-9, caspase-8, and Bcl-2 in the HNSCC cells with MUC1 overexpression or downexpression following by IR exposure. The results demonstrated that MUC1 overexpression inhibited IR-induced caspase-3, caspase-9, and caspase-8 expression and upregulated bcl-2 expression, and was resistant to IR-induced cell apoptosis. However, targeting MUC1 blocked IR-induced caspase-3, caspase-9, and caspase-8 upregulation and downregulated bcl-2 expression, and was resistant to IR-induced cell apoptosis.

Irradiation (IR) can cause DNA damage, such as base lesions, DNA-protein cross-links, single-strand breaks, and DSBs, resulting in cell death or genomic instability that could lead to carcinogenesis [29]. Phosphorylation of histone H2AX on serine 139 (γH2AX) is an early step in cellular response to DNA damage, when the damage results in the formation of a double-strand break (DSB). Enhanced DSB repair is an important mechanism by which cells may be comeresistant to IR [30]. The γH2AX fluorescence assay became one of the methods of choice for the detection of radiation-induced DSB [31]. Furthermore, γ-H2AX has become a powerful biomarker for the quantification of DSB levels in cells and tissues [32]. 53BP1 is a p53 binding protein of unknown function that binds to the central DNA-binding domain of p53 and mediates DSB repair, checkpoint signaling, and the synapsis of DSB ends [33–35]. Considering the genuine functions of γH2AX and 53BP1 in DSB repair, the simultaneous analysis of γH2AX and 53BP1 by immunofluorescence microscopy may be a useful method for the precise analysis of the formation and repair of DSB. In the study, MUC1 overexpression increased γH2AX- and 53BP1-positive foci in the IR treated cells, and targeting MUC1 decreased γH2AX- and 53BP1-positive foci in the IR treated cells. These data support our hypothesis that MUC1 is associated with DNA DSB repair mechanisms following by IR treatment, and MUC1 overexpression is a major contributing factor toward radioresistance.

## Conclusion

MUC1 enhances DSB repair and resistance to IR-induced apoptosis in HNSCC cells. Targeting MUC1 increases radiation sensitivity of HNSCC cells by enhancing irradiation-induced apoptosis and hampering DNA DSB repair. Thus, MUC1 may represent a target to improve multimodal therapeutic outcome of HNSCC.

## Highlights

Enforced MUC1 expression enhances radioresistance in HNSCC cells.

Targeting MUC1 expression enhances radiosensitivity *in* HNSCC cells.

Enforced MUC1 expression induced radioresistance is associated with enhanced DSB repair in HNSCC cells.

Targeting MUC1 enhanced radiosensitivity is associated with decreased DSB repair *in* HNSCC cells.
